# “French Phage Network”—Second Meeting Report

**DOI:** 10.3390/v9040087

**Published:** 2017-04-21

**Authors:** Clara Torres-Barceló, Oliver Kaltz, Rémy Froissart, Sylvain Gandon, Nicolas Ginet, Mireille Ansaldi

**Affiliations:** 1 Plant Populations and Bio-aggressors in Tropical Ecosystems (PVBMT) UMR, Pôle de Protection des Plantes, F-97410, Saint-Pierre, Reunion Island, France; clara.torres@univ-montp2.fr; 2 Institut des Sciences de l'Evolution, UMR 5554 (CC065), Université de Montpellier, F-34095 Montpellier, France; oliver.kaltz@umontpellier.fr; 3 CNRS, IRD, Université de Montpellier, Laboratory «Maladies Infectieuses & Vecteurs : Ecologie, Génétique, Evolution & Contrôle» (MIVEGEC), UMR 5290, F-34394 Montpellier, France; remy.froissart@cnrs.fr; 4 CNRS, Université de Montpellier, Centre d'Ecologie Fonctionnelle et Evolutive (CEFE), UMR 5175, F-34293 Montpellier, France; sylvain.gandon@cefe.cnrs.fr; 5 CNRS, Aix-Marseille Université, Laboratoire de chimie bactérienne, UMR 7283, Institut de Microbiologie de la Méditerranée, F-13402 Marseille, France; nginet@imm.cnrs.fr or ansaldi@imm.cnrs.fr

**Keywords:** phage, bacteria, infection, co-evolution, virulence, resistance, phage therapy, structural biology, genomics, France

## Abstract

The study of bacteriophages (viruses of bacteria) includes a variety of approaches, such as structural biology, genetics, ecology, and evolution, with increasingly important implications for therapeutic and industrial uses. Researchers working with phages in France have recently established a network to facilitate the exchange on complementary approaches, but also to engage new collaborations. Here, we provide a summary of the topics presented during the second meeting of the French Phage Network that took place in Marseille in November 2016.

## 1 Introduction

In 2017, we celebrate the centennial of the founding paper for bacteriophage research by Felix d'Hérelle [1]. Bacteriophages, the viruses of bacteria (phages, hereafter), are currently in the scientific and public focus, due to their biotechnological implications (notably the CRISPR-Cas system) [2,3], but also due to the renewed interest in their therapeutic application as an alternative or adjuvant to antibiotics to deal with the threatening emergence of multi-drug resistant bacteria [4,5]. Furthermore, metagenomic approaches have radically improved our knowledge of the ecology of phages found in highly diverse environments, such as the ocean or the mammalian gut (e.g., [6,7,8]). At the same time, new technologies refining image resolution (such as cryoelectron microscopy or particle accelerators) have made it possible for structural biologists to provide highly accurate molecular models of phage proteins and structures (e.g., [9,10,11]). While important progress is being made in these different areas, the world phage research community tends to be scattered in different scientific societies and conferences (molecular biology, structural biology, evolution, or clinical microbiology, for example) and as a consequence hardly interacts. To overcome this obstacle in our community, the French Phage Network was launched in 2015. This network gathers researchers from highly diverse areas of phage research and aims to create new synergies between disciplines, from basic research to biotechnologies and therapeutic applications.

After a first inaugurating meeting in Montpellier in 2015, the second meeting of the French Phage Network was held in November 2016, at the Laboratoire de Chimie Bactérienne (Centre national de la recherche scientifique-Aix-Marseille Université (CNRS-AMU)) in Marseille. This conference (“Phages on MARSeille”) brought together an enjoyable mix of researchers working in the different disciplines of phage research in France, but also included several colleagues from the UK, Germany, Belgium and Canada (see Supplementary file S1 for a full list of the attendees). These yearly meetings bring together young and consolidated scientists working with complementary approaches, and aim to provide an interface between French public research institutions, hospital clinicians and the private sector. In this article, we present an overview of the presentations given at the meeting, keeping in mind that even more results and projects were presented during poster sessions (Table 1). The meeting was organised in three sessions dealing with research in the fields of (i) Molecular Interactions; (ii) Evolution and Ecology; and (iii) Therapy.

## 2. Summary of Scientific Sessions

### 2.1. Host–Phage Molecular Interactions

**Figure 1:**
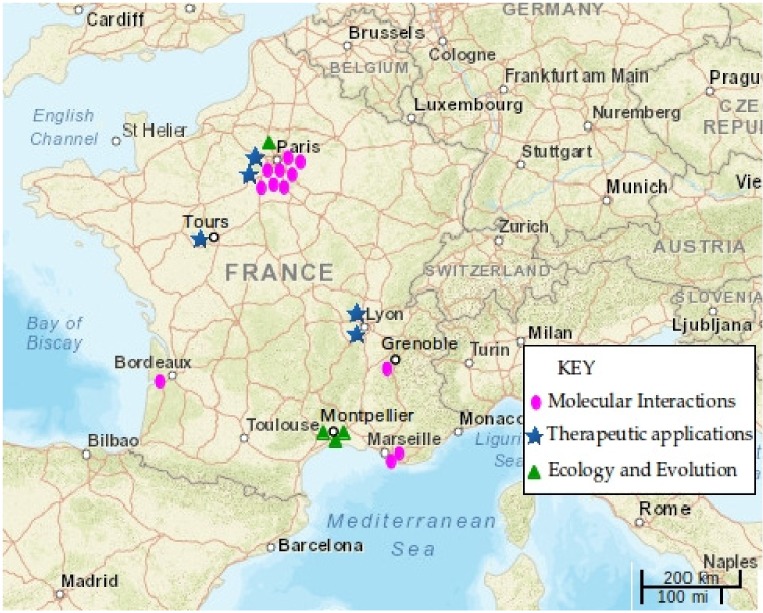
Geographic location of research groups presenting their work at the “Phages on MARSeille” meeting in 2016. Symbols and colours indicate the scientific or applied approach of each research. Map created with [14].

This session dealt with approaches related to molecular interactions between phages and their bacterial hosts, with highly diverse contributions from research teams from all around France (Figure 1). For example, structural analysis can provide key information about the infection mechanism of phages, as shown by Christian Cambillau. His team studies the structure and function of phage baseplates, tail components of phages from the *Siphoviridae* family involved in host recognition and attachment. X-ray crystallography and electron microscopy provided new insights into the role of baseplates in the phage life-cycle during infection of the Gram-positive bacteria *Lactococcus lactis* [12,13]. Present investigations address the similarity of mechanisms of baseplate activation across different *Siphoviridae* phages. In the long term, these studies should be of great help for the dairy fermentation sector where regular phage attack against lactic bacteria causes considerable economical loss.

Several talks reported on the interaction between the virulent phage T5 (*Siphoviridae*) and the Gram-negative bacteria *Escherichia coli*. Phage T5 injects its DNA genome by way of a 2-step mechanism, with initially only 8% of its genome injected. Early expression of phage genes is crucial to hijacking and controlling the metabolism of the host *E. coli* before the transfer of the remaining DNA resumes. Charles-Adrien Arnaud showed in detail how the structure and properties of the tail tube protein of T5 can be investigated by combining different structural methods (X-ray crystallography, nuclear magnetic resonance spectroscopy and electron microscopy). This type of approach allows the study of interactions that are generally difficult to detect through genome sequencing or other comparative methods [15]. Leo Zangelmi presented his work on the functional characterization of the early expressed *a1* and *a2* genes, which are part of the initially injected phage genome. A1 and A2 proteins are essential for the completion of the phage DNA transfer and have been found to be conserved in all T5 genomes sequenced so far. While A1 degrades the host DNA, A2 may be a DNA-binding protein that increases the activity of A1 and may have important regulatory functions. The overall aim of the team is to understand the structure and mechanistic role of all T5 conserved early proteins [16,17].

Audrey Labarde outlined the spatio-temporal organization of the infection by a different *Siphoviridae*, the virulent phage SPP1. The replication mechanism and assembly of this phage in its host, *Bacillus subtilis*, have been thoroughly explored by the team, through the use of genetic, structural and biochemistry approaches [18,19,20]. The speaker showed how phage DNA is massively produced in defined viral replication factories within the cytoplasm. Paulo Tavares, from the same research team, further reviewed several studies on the mechanism of DNA packaging of the SPP1 phage. In this and other phages, the synthesised DNA molecule is cut when a precise quantity of DNA is reached inside the capsid (headful cleavage). By using mutagenesis and hybrid oligomers, the researchers elucidated the functional details of the SPP1 portal protein, the sensor, and the basis of the DNA head filling mechanism [19,20]. 

Another approach to study the gradual changes during the phage life cycle was taken by Anne Chevallereau, who investigated the transcriptional strategies of two virulent therapeutic phages infecting the Gram-negative *Pseudomonas aeruginosa*: PAK_P3 and PAK_P4 (both from the *Myoviridae* family). She found different patterns of temporal regulation of the viral gene expression as well as the modification of the host gene transcription levels. The comparison between transcriptomes not only provides information on the precise interaction between phages and bacteria, but also indicates to what extent infection mechanisms are conserved between phages [21]. 

Genomic and biochemical analyses of phages were also presented at the meeting. Anne Jamet showed how metagenomic analyses can yield valuable information on the complex interplay between bacteria and phages. Her team identified the MuF domain as highly abundant in phage genomes. However, when associated with a toxin domain, MuF was restricted to temperate ones. It is hypothesised that MuF-toxins play a role in the competition of the phage host with other bacteria or in manipulating the host metabolism, as described for other toxins [22], but also in the lysis-lysogeny decision during the life-cycle of these temperate phages. François Lecointe reported on the biochemical characterization of a Sak4 protein, a recombinase encoded by the temperate phage HK620 (*Podoviridae*) infecting a specific *E. coli* strain (TD2158). Interestingly, the Sak4 enzymes are RecA paralogs related to the archaeal RadB recombinases. Sak4 from HK620 has been found to be the first single strand annealing protein requiring ATP and using a single-strand DNA-binding protein (SSB) to enhance its activity. This team has a general interest in recombinases, as they are key enzymes involved in genome repair and replication [23], as well as in bacterial genome remodelling via phage recombination [24].

A new perspective regarding the identification and quantification of phages was provided by Martine Boccara. Her team has developed an optical method for nanoparticle analysis, allowing the differentiation between viruses and vesicles [25]. The technique is based on measurements of light scattering together with the Brownian motion, from which the diameter of nanoparticles can be calculated. This method was used to analyse the distribution of vesicles and viruses in different ocean water samples from the Tara Oceans project, an expedition set out to describe plankton and other microorganism diversity at a planetary scale [26].

Several presentations were at the interface between fundamental and applied research. Raphaëlle Delattre investigated endotoxin release by bacteria when lysed by phages. Studying such effects is crucial for the evaluation of the risk of pro-inflammatory responses in patients receiving a phage therapy treatment against a bacterial infection. The virulent phages studied were 536_P1 and LM33_P1 (*Myodoviridae* and *Podoviridae*, respectively), attacking two clinical strains of *E. coli* (536 and LM33) [27,28]. The results showed that the level of the endotoxin LPS (lypopolysaccharide) released after exposure to phages was comparable to that observed when using antibiotics [29]. This suggests that the risk of therapeutic use of these phages is at least not higher than that of an antibiotic treatment.

Laurent Debarbieux presented an experimental evolution study on the evolutionary potential of therapeutic phages, using a mouse model system. In an in vivo experiment, the team analysed the population metagenomics of phage P10 in mice infected with an invasive strain of *E. coli* (LF82) [30]. They found that the phage evolved a wider host range, notably for the commensal MG1655 *E. coli*. The genetic change underlying this host jump consists of a single amino-acid change in a tail fibre protein. However, surprisingly, host jumps did not occur in in vitro or in axenic mice experiments. These results have important implications for the assessment of unwanted 'evolutionary side effects' of phage therapy, and they highlight the need for molecular studies to consider different environmental conditions.

Finally, Cécile Philippe described her work on the microbiological diversity of wine and grapes. Her team investigates phages attacking the lactic acid Gram-positive bacterium *Oenococcus oeni* [31,32], and more specifically the impact of polyphenolic compounds present in wine on the infection rate by the virulent oenophage ΦOE33PA (*Siphoviridae*). By lysing these bacteria, phages can affect the malolactic fermentation (MLF) process and thus the wine making process. However, when MLF is not desired (white wines), such phages could represent valuable biocontrol agents. Deeper understanding of the interplay between fermentation and phage life cycle is, however, still needed before such research may be translated into biotechnological applications.

### 2.2. Therapeutic Applications of Phages

In this session, practical aspects of using phages as therapeutic agents against pathogenic bacteria being developed in France were discussed (Figure 1). Alain Dublanchet, a pioneer in the recent use of phage therapy in France, reported on selected cases of successful phage treatments. Patients suffering from antibiotic resistant chronic infections (e.g., *Staphylococcus aureus*) were treated with commercially available phage cocktails from Russia and Georgian Republic. Typically, phage treatment was effective and even full recovery was observed in patients after one or several applications, in certain cases saving patients from already scheduled amputations. As a future perspective, the speaker suggested that phage cocktails could be produced on demand, by the pharmacy service of a hospital. As a matter of fact, in the past and sometimes still today, these pharmacies prepare antibiotic cocktails and other personalised preparations.

Clinical trials are indispensable to evaluate the general efficacy of phage therapy. Olivier Patey presented current projects concerning phage therapy in France, such as Phagoburn (see below) and PHOSA [33]. The recently launched PHOSA project aims to develop a phage cocktail effective against bone, joint and diabetic foot ulcer infections, caused by *S. aureus* and *S. epidermidis*. Plans were outlined for the creation of reference structures to organize and standardise phage treatments in the EU, as promoted by the association “Phagothérapie 2020”. This plan would require coordination between hospitals, phage banks and phage producing reference centres as well as associated research groups.

Interesting insights into the Pneumophage project were provided by Cindy Fevre [34]. Like the Phagoburn and PHOSA projects, Pneumophage is a public–private initiative involving the company Pherecydes Pharma, public research institutions (such as INSERM), and hospitals. A detailed account was given on the criteria, methods and results obtained in the development of a phage cocktail aimed at treating infections of the respiratory tract produced by the opportunistic and often multi-resistant *P. aeruginosa*. Host range, characteristics of phages in a nine-phages cocktail, efficiency and manufacturing aspects (such as stability of the preparation), were considered. The effect of the phage administration route, and a comparison with regularly administrated antibiotics were evaluated in vivo, using mice. 

François Ravat, a medical doctor and collaborator with Pherecydes Pharma, presented the current state of the Phagoburn project [35]. This clinical assay of phage therapy involves three European countries (Belgium, Switzerland and France) and eleven hospitals. The purpose is to evaluate the efficacy of topical application of a phage cocktail against skin infections by *P. aeruginosa* in severely burned patients [36,37]. This very exciting trial is based on a double-blind method and compares phage therapy with the standard chemical treatment. Due to European health regulations, results can only be made public after the end of the trial (expected during the first semester of 2017), but the speaker hinted at successful results being released soon.

Mai Huong Ly-Chatain, a researcher from a private company, gave an extensive overview of the possible factors influencing phage therapy successes and failures [38]. As illustrated by various examples, treatment success likely depends on the dose of phage, timing and administration route, accessibility to the bacteria, as well as the risk of neutralization of phages by the patient's immune system or by unfavourable environmental conditions. Based on these considerations, recommendations for the successful use of phage therapy were formulated

### 2.3. Ecology and Evolutionary Research of Phages

In this session, two talks discussed the CRISPR-Cas adaptive immune system of prokaryotes from an evolutionary perspective. CRISPR-Cas is a highly specific mechanism of defence against viruses, widely used by bacteria and archaea [39]. The system is based on the excision of small regions (called “spacers”) derived from phage genomes and their insertion into arrays contained in the prokaryotic genome. These spacers will subsequently guide the defence against specific phages. First, Stineke van Houte presented experimental evidence showing that the ability of phages to evade CRISPR-Cas immunity depends on the number and diversity of spacers present in the host genome [40]. In her experiment, the level of diversity was artificially manipulated to generate different bacterial populations (*P. aeruginosa* or *Streptococcus thermophilus*) with increasing numbers of spacers. These populations were then exposed to phages (DMS3vir and 2972, respectively) and the viral densities were monitored over time. Phages were only able to persist in the population when the diversity of spacers present in the bacterial genome was low. The ability of CRISPR-Cas immunity to generate diversity and to prevent phage escape may explain the emergence of sophisticated anti-CRISPR mechanisms in some phages [40,41,42].

Hélène Chabas investigated the adaptation of the virulent phage 2972 to the CRISPR-Cas defence system in *S. thermophilus*, combining theoretical modelling approaches and experimental evolution. In particular, increased bacterial resistance (induced by the addition of a single spacer in CRISPR-Cas) affected the rate of emergence of escaping phages. Different resistant bacteria (all carrying a single, but different, spacer) varied in their ability to prevent phage evolution. This is likely due to the variability in fitness costs associated with escape mutations in the phage but further experimental work is needed to confirm this hypothesis.

When therapeutic phages are used to treat bacteria, it is necessary to evaluate the evolvability (capacity for adaptive evolution) of these bacteria to resist the phages. Julien Lossouarn presented a complete analysis of the interaction between the Phi29-related virulent phage "Idefix" (*Podoviridae* family) and the Gram-positive bacterium *Enterococcus faecalis*. The rate of spontaneous resistant mutants to the phage was determined and the mechanistic basis of resistance characterised. Most resistant bacteria exhibited modified cell wall polysaccharides, which serve as phage receptors. In other cases, resistance was due to the presence of a prophage-encoded membrane protein conferring superinfection exclusion. These observations suggest that the phage Idefix may not be a useful therapeutic phage, but other phages may provide good alternatives. In addition, lysogeny was confirmed as an important parameter to consider for phage therapy, as it sometime interferes with infection by virulent phages.

Phage therapy may be used in combination with antibiotics to control pathogenic bacteria [43,44], but we still know little about the evolutionary constraints induced by such combined therapies, namely the potential of bacteria to evolve resistance to either antibiotics and/or phages. Building on previous studies [45,46], two presentations dealt with this topic. Clara Torres-Barceló showed preliminary data from experiments testing for generalities in the patterns of population dynamics and resistance evolution under combined antibiotic-phage in vitro treatments. By employing a panel of antibiotics (ceftazidime, ciprofloxacin and erythromycin) and phages (LKD16, LUZ7, 14/1, EL: two *Podoviridae* and two *Myoviridae*, respectively) individually or in combination against *P. aeruginosa*, combination treatments moderately decreased bacterial population density and limited antibiotic resistance levels, compared to treatments with only antibiotic or phage. Unexpectedly, there were no significant differences between the fitness (measured as replication capacity) of phages and bacteria evolved in combined or single treatments in the long term (60 bacteria generations).

Shifting focus to more short-term effects, Mireille Ansaldi reported on a study evaluating the effects of low concentrations of various antibiotics for the proliferation of phage T5 attacking *E. coli*. In this case, an increase in the production of phage particles was observed in the presence of certain antibiotics (namely beta-lactams, but also chloramphenicol and spectinomycin). These results corroborate previous findings of synergistic effects between phages and antibiotics, but also give more insights into the mechanisms underlying this interaction [43,47]. As expected, bacteria surviving the combination treatments had lower levels of resistance to both antibiotics and phages, but also showed a surprising reduction in the frequency of mutagenesis.

A conceptual and methodological talk concluded this session. Oliver Kaltz proposed a simple and robust statistical method to infer the rate of antagonistic co-evolution between phages and bacteria in 'time-shift assays', measuring phenotypic changes in bacterial resistance and phage infectivity over multiple generations. Typically, such studies only provide separate measures of evolutionary change in bacteria and phages [48], whereas here, a single combined co-evolution score is proposed. This new summary statistic can facilitate comparisons between experimental treatments [49], and may serve as a metric for meta-analyses of coevolution across different studies.

## 3. Conclusions and Perspectives

Over the two days of the meeting, talks given by researchers from private and public institutions, as well as by medical doctors, covered a broad diversity of approaches on phage research. This provided the unique opportunity for discussions between scientists working on similar systems, but asking different questions and/or using different approaches. New insights can result from such scientific crosstalk and common research questions and complementarities can be identified. Indeed, one of the main aims of the French Phage Network is to provide communication platforms (through the meeting or discussion groups), from which, ideally, new collaborative projects may arise. We have seen, for instance, how molecular aspects of phage–bacteria interactions have important consequences for the ecological dynamic of the microbiomes, and ultimately should become of interest for medical purposes. This interplay between fundamental and applied spheres is particularly relevant in the context of the therapeutic use of phages. The meeting thus provided an exciting update on the current state-of-the-art of the clinical use of phages in France and in Europe. It outlined the still severe constraints imposed by the legal framework of phage therapy in Europe [50,51,52], which renders the production of phages very difficult. Both clinicians and researchers emphasised the urgent need for an appropriate legislation for the use of phages and they outlined different specific propositions to do so. Clarification of the legal situation may also facilitate the funding of collaborations between fundamental researchers and more clinically oriented institutions. 

As illustrated in this report, the French research in the field covers a wide variety of perspectives and microorganisms (Table 1, Figure 1). However, we also note that a large fraction of the presented experimental research used only two bacterial model systems: *E. coli* and *P. aeruginosa* (11 out of 19 presentations). We hope that, in the near future, the network will reach researchers working with other phage–bacteria systems or even using additional approaches. For example, the workshop and network could benefit from associations with industrial applications of phages, communication with medical doctors, theoretical approaches, or more studies about natural phage communities. The next meeting is scheduled on November 20–21, 2017, at the Institute for Integrative Biology of the Cell (Université Paris-Sud, Orsay, France). To contact the French Phage Network, an email address is available (contact@phages.fr) while the website (www.phages.fr) is being built.

## Figures and Tables

**Table 1:** Posters presented during the French Phage Network 2016 meeting.

Poster Title	Presenter
«Accessing virus genomes out of metagenomics data: Improving statistical and bio-informatics analytic tools to better assess the contribution of phages to microbial ecosystems»	Stéphane Chaillou
«Proliferation of phage K in the raw milk in the presence of protease or cation 2+»	Mai Huong Chatain-Ly
«Host factors involvement in prophage maintenance in *E. coli*»	Maëlle Delannoy
«On-site detection of bacterial pathogens using phage-based light-emitting biosensors»	Nicolas Ginet
«Bacterial vaginosis: Are bacteriophages involved in dysbiosis of human genital tract?»	Rémy Froissart
«Characterization of two Leptospira lytic bacteriophages»	Olivier Schiettekatte
«Evaluation of alternative animal models for testing the efficacy of phages as therapeutic agents»	Catherine Schouler
«BcepMu/B3-like transposable prophages in proteobacteria and pseudomonads»	Ariane Toussaint
«Félix d’Hérelle Reference Center for Bacterial Viruses»	Denise Tremblay

## Supplementary Materials

The following are available online at www.mdpi.com/1999-4915/9/4/87/s1, Supplementary file S1: Affiliations of attendees.
