# Supplementary material for: “French Phage Network”—Second Meeting Report
Source: Viruses. 2017 Apr 21;9(4):87. doi: 10.3390/v9040087 (PMC5408693; doi:10.3390/v9040087)
Supplement: Supplementary file 1 [file viruses-09-00087-s001.docx]

Affiliations of attendees

**Surname Name Affiliation**

Ansaldi Mireille Laboratoire de Chimie Bactérienne, Marseille

Arnaud Charles Institut Biologie Structurale, Grenoble

Auzat Isabelle Institute for Integrative Biology of the Cell, Gif/Yvette

Battesti Aurélia Laboratoire de Chimie Bactérienne, Marseille

Bastien David Sysmex Europe GmbH, Hamburg, Germany

Bernardet Maude Journaliste

Berngruber Thomas PTC GmbH, Hamm, Germany

Boccara Martine ISYEB mnhn, Paris

Boulanger Pascale Institute for Integrative Biology of the Cell, Orsay

Boulon Pascal Université de Lyon

Bourhy Pascale Institut Pasteur, Paris

Breyton Cécile Institut Biologie Structurale, Grenoble

Cambillau Christian Architecture et Fonction des Macromolécules Biologiques, Marseille

Chabas Hélène CEFE-CNRS, Montpellier

Chaillou Stéphane INRA MICALIS, Jouy-en-Josas

Champ Stéphanie Laboratoire de Chimie Bactérienne, Marseille

Chatain-Ly Mai Huong ISARA, Lyon

Chevallereau Anne Institut Pasteur, Paris

Clavijo Fernando Laboratoire de Chimie Bactérienne, Marseille

Cornuault Jeffrey INRA MICALIS, Jouy-en-Josas

Corral Lugo Andres Institute for Integrative Biology of the Cell, Gif/Yvette

d'Humieres Camille Institut Pasteur, Paris

De Paepe Marianne INRA MICALIS, Jouy-en-Josas

Debarbieux Laurent Institut Pasteur, Paris

Delannoy Maëlle Laboratoire de Chimie Bactérienne, Marseille

Delattre Raphaelle Institut Pasteur, Paris

Dion Moïra Université de Laval, Canada

Dublanchet Alain Centre hospitalier de Villeneuve-Saint-Georges

Dugat-Bony Eric INRA, Thiverval-Grignon

Dupressoir Anne INSERM, Paris

Erauso Gaël Aix-Marseille Université, Marseille

Fevre Cindy Pherecydes Pharma

Froissart Rémy MIVEGEC-CNRS, Montpellier

Gandon Sylvain CEFE-CNRS, Montpellier

Gautheret-Dejean Agnès Laboratoire de Virologie, Hôpital Pitié Salpétrière, Paris

Ginet Nicolas Laboratoire de Chimie Bactérienne, Marseille

Jamet Anne INSERM - INEM, Paris

Kaltz Oliver ISEM, Montpellier

Labarde Audrey Institute for Integrative Biology of the Cell, Gif/Yvette

Le Hénaff Claire I.S.V.V. - Université de Bordeaux, Bordeaux

Lecointe Francois INRA MICALIS, Jouy-en-Josas

Lossouarn Julien INRA MICALIS, Jouy-en-Josas

Marques Godinho Lia Institute for Integrative Biology of the Cell, Gif/Yvette

Mathieu Aurélie INRA MICALIS, Jouy-en-Josas

N'guetta Eric Neovia, Saint-Nolff

Patey Olivier CHI Lucie et Raymond Aubrac, Paris

Petit Marie-Agnès INRA MICALIS, Jouy-en-Josas

Petitjean Charlotte Pherecydes Pharma, Romainville

Philippe Cécile I.S.V.V. - Université de Bordeaux, Bordeaux

Philippot Hélène LMCPA - Université de Valenciennes Hainaut Cambresis

Quillet Laurent Université de Rouen, Rouen

Ramirez Luis Institute for Integrative Biology of the Cell, Orsay

Ravat François Centre des brulés, Lyon

Rebaudet Stanislas Médecin, Marseille

Regeard Christophe Institute for Integrative Biology of the Cell, Orsay

Rossier Ombeline Institute for Integrative Biology of the Cell, Orsay

Schiettekatte Olivier Institut Pasteur, Paris

Schouler Catherine INRA, Nouzilly

Spinelli Silvia Architecture et Fonction des Macromolécules Biologiques, Marseille

Richard Fabrice Quattrocento, Paris

Tavares Paulo Institute for Integrative Biology of the Cell, Gif/Yvette

Torres-Barceló Clara CIRAD, La Réunion

Touchon Marie Institut Pasteur, Paris

Toussaint Ariane Université Libre de Bruxelles, Belgique

Tremblay Denise Université de Laval, Canada

Trotereau Angélina INRA, Nouzilly

van Houte Stineke University of Exeter, UK

Westra Edze University of Exeter, UK

Zangelmi Léo Institute for Integrative Biology of the Cell, Orsay
